# Remarks on the Chemical Theory of Diet

**Published:** 1848-01

**Authors:** R. Dick


*Remarks on the Chemical Theory of Diet.* By. R. Dick, M.D. Without at all seeking to question the ingenuity, accuracy, and importance of the late chemical researches of Liebig and others, into the nature and ultimate uses of diet, we may be allowed to observe, that, practically, the results which, according to their theories, ought to be witnessed, are far from being so universally.

In the Pampas of South America there is a numerous race who for weeks, months, and even years, eat nothing but nitrogenous food, without any intermixture of respiratory or calorifiant. These are the Gauchos—men who not only are of the most vigorous and active habits. but are, perhaps, the healthiest men of the world. Their solid aliment consists exclusively of dried cow-flesh: their drink is water: they use no bread or vegetables. So much, then, for an example of human beings living, and living in full health, on a diet exclusively nitrogenous.

Captain Abbott, also, in his Travels from Heraut to Khiva, informs us that the Kurrauks, a tribe of Tartars, live in summer "almost *solely* upon the milk of their camels, mares, and sheep, *without bread or vegetables*, knowing only at long intervals the luxury of flesh; but, as the winter pasture can furnish but a scanty supply of milk, they kill, at the commencement of winter, all their old camels, horses, and sheep, and salt them, as a winter store. *These are eaten without any accompaniment of bread or vegetable.*"—Vol. ii. Appendix, pp. 45–6. Yet these are among the healthiest and hardiest of our species.

Now, for an example of an opposite kind, we might look no further, for illustration, than Ireland, where millions of men have lived, and lived in robustness, on an aliment almost wholly, if not wholly, destitute of nitrogenous principles, and certainly altogether wanting in formed *fibrin*. In India and in China, large masses live, almost as much from choice as from necessity, on rice, which they prefer to every other species of grain, not excepting wheat. These facts are practically at variance with Liebig's theory and the alleged necessity of a mixed diet, and make it probable, if not certain, that the death or disease which has followed the sudden change from a mixed diet to one exclusively animal or exclusively vegetable, has been rather due to the abruptness of the change, than to the change itself. By degrees, eagles have been taught to live on grain; pigeons on flesh.—*London Med. Gaz.—Southern Jour. Med. and Pharmacy.*

